# Coronavirus disease 2019-associated encephalitis and concomitant subdural hematoma: a case report

**DOI:** 10.1186/s13256-026-06148-y

**Published:** 2026-06-03

**Authors:** Jin Liu, Wenmian Huang, Xuqing Wu, Yu Ma

**Affiliations:** Department of Neurology, Zhongshan Hospital, Fudan University, 180 Fenglin Road, Shanghai, 200032 People’s Republic of China

**Keywords:** COVID-19, Central nervous system, Encephalitis, Subdural hematoma, Case report

## Abstract

**Background:**

Coronavirus disease 2019 (COVID-19), induced by severe acute respiratory syndrome coronavirus 2 (SARS-CoV-2), presents a global pandemic with evolving viral variants. In addition to respiratory symptoms, a growing trend of reports indicates that the central nervous system could also be affected in COVID-19 patients.

**Case presentation:**

Herein, we reported a case of a 61-year-old Chinese male with fever, psychiatric symptoms, and concomitant subdural hemorrhage. Although naso-oropharyngeal swab tests for SARS-CoV-2 ribonucleic acid detections were negative, the metagenomic next-generation sequencing from cerebrospinal fluid (CSF) samples showed the exclusive positive finding of SARS-CoV-2. The patient was diagnosed with probable COVID-19-associated encephalitis, and was recovered after receiving anti-infection medications, high-dose methylprednisolone pulses (1 g/day for 5 days), and subsequent intravenous immunoglobulin (0.4 g/kg body weight for 5 days) therapies.

**Conclusion:**

Our case underscores the importance that for patients with fever and unexplained neuropsychiatric symptoms, it is recommended to conduct CSF testing to screen for possible pathogen infections, and to perform cranial imaging promptly to detect concomitant lesions.

## Introduction

Coronavirus disease 2019 (COVID-19), an infectious disease induced by severe acute respiratory syndrome coronavirus 2 (SARS-CoV-2), could affect multiple organs and systems. Neurological manifestations associated with COVID-19 have also been reported, including hypoxic‐ischemic brain injury, encephalitis, and neuromuscular dysfunction [1]. Specifically, encephalitis could lead to severe outcomes and significantly increase the mortality rates [2]. Therefore, timely identification of COVID-19-associated encephalitis is essential for clinicians. Herein, we reported a case of a Chinese male presenting with fever, abnormal psychiatric behaviors, and concomitant subdural hematoma. There were little evidences of SARS-CoV-2 infection in initial examinations. Further metagenomic sequencing supported the diagnosis of probable COVID-19-associated encephalitis. The case highlights the importance of detecting potential pathogen infections in cerebrospinal fluid (CSF) samples and performing cranial imaging promptly to identify concomitant brain lesions.

## Case presentation

**Fig. 1:**
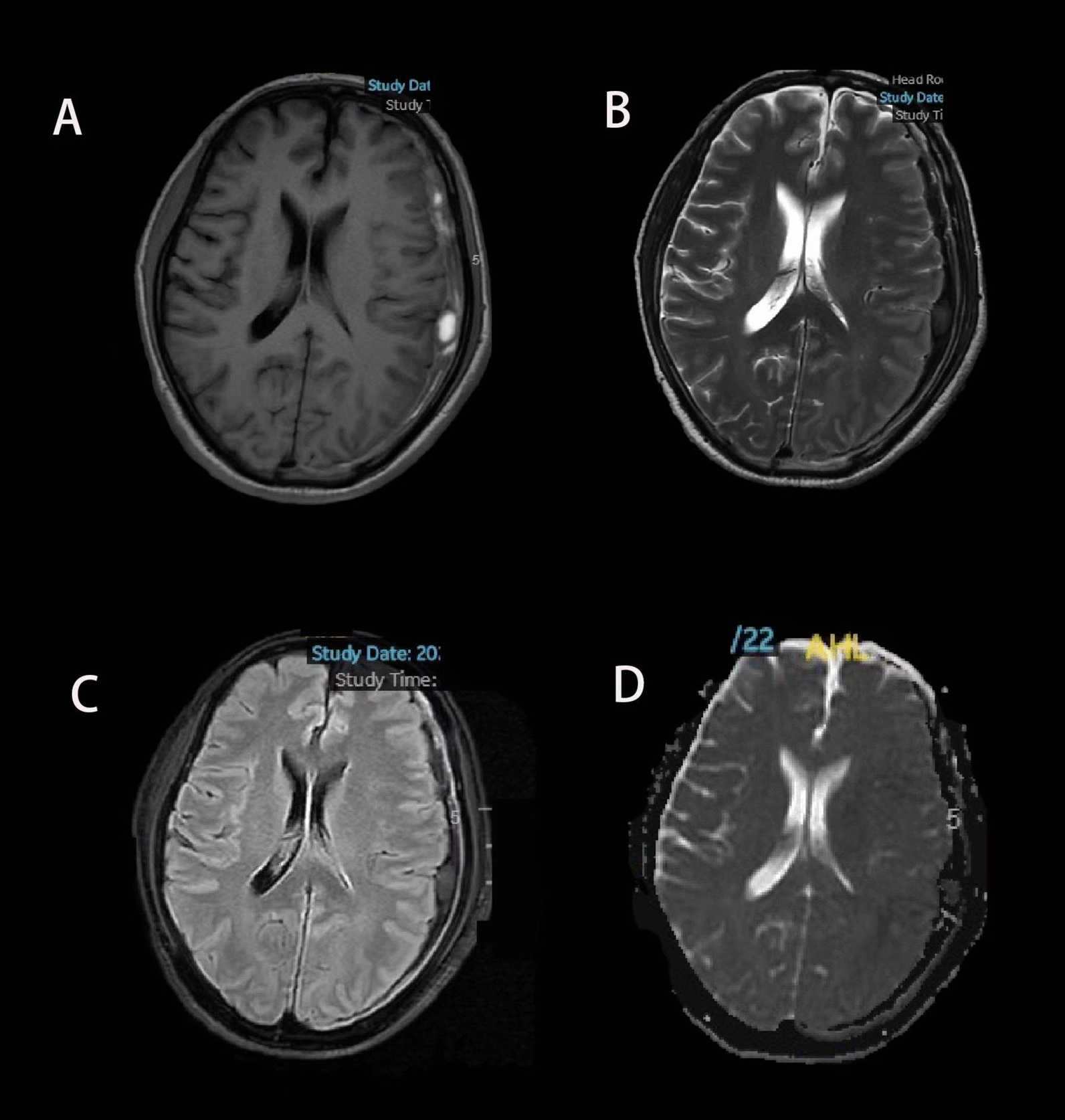
Magnetic resonance imaging of the brain on admission. Axial T1-weighted (**A**), T2-weighted (**B**), T2-weighted fluid attenuated inversion recovery (**C**), and apparent diffusion coefficient (**D**) sequences of the brain magnetic resonance imaging (MRI) on admission revealed left subdural hemorrhage

A 61-year-old Chinese male first suffered from a fever, and was admitted to community hospital for probable upper respiratory tract infection. On the 5th day, he presented abnormal psychiatric symptoms including disorganized speech, delirium, hostility toward family members, and even brief episodes of striking the head against the ground. This patient was then transferred to our hospital. Before admission, the long-term medications the patient took were anti-hypertensive and anti-diabetic drugs (enalapril and metformin), with no anticoagulant or antiplatelet drugs. He and his immediate family members had no past history of psychiatric disorders. On admission, the temperature of the patient was 37.9 °C, and other vital signs were normal. Positive neurological symptoms included cognitive confusion, memory deficits, and diminished language function, along with involuntary tremors in bilateral upper extremities. Laboratory test results were as follows: C-reactive protein (CRP) 16.8 mg/L, neutrophil percentage 77.0%, platelet count 185*10^9/L, international normalized ratio (INR) 1.05, prothrombin time (PT) 12.6 s, activated partial thromboplastin time (APTT) 27.3 s, and D-dimer 4.70 mg/L. Blood tests also revealed elevated levels of SARS-CoV-2 immunoglobulin (Ig) G antibodies (139.30 AU/mL), interleukin (IL)−6 (6.8 pg/mL), and tumor necrosis factor (TNF) (10.1 pg/mL). Other parameters, including leukocytes, serum electrolytes, complete blood count, erythrocyte sedimentation rate (ESR), and liver and kidney function, were within normal ranges. Meanwhile, routine blood culture and SARS-CoV-2 ribonucleic acid detection via naso-oropharyngeal swab yielded negative results. Chest computed tomography (CT) imaging revealed no obvious inflammatory foci in either lung. However, brain CT images demonstrated a left subdural hematoma appearing as a crescent-shaped area of high density. The major axis, minor axis, and number of lesion layers were 12 cm, 1.3 cm, and 20, respectively, with a layer thickness of 5 mm. Brain magnetic resonance imaging (MRI) (Fig. 1) further suggested that the subdural hematoma might be surrounded by an edematous band. His electroencephalogram (EEG) monitoring showed diffused 3–6 Hz slow wave discharges with low amplitudes symmetrically in both hemispheres. Given the presence of a left subdural hemorrhage, lumbar puncture was deferred. Based on the clinical presentation and examination findings, intracranial infection was considered the primary diagnostic possibility. The patient was empirically treated with ganciclovir (0.25 g/q12h), vancomycin hydrochloride (1 g/q12h), and meropenem (1 g/q12h). On the 10th day, the patient’s psychiatric symptoms persisted, and body temperature remained elevated at approximately 38 °C.

On the 15th day, a lumbar puncture was performed. The CSF pressure was 120 mmH_2_O, and the relevant CSF examination findings are summarized (Table 1). Other CSF tests including direct smear examination (bacteria, fungi, Cryptococcus, and acid-fast bacilli), culture examination (bacteria, mycobacteria, and fungi), nucleic acid testing of tuberculosis bacillus, and antibodies against autoimmune encephalitis and paraneoplastic syndrome were all negative. However, metagenomic next-generation sequencing (mNGS) of the CSF sample yielded a positive result exclusively for SARS-CoV-2 with 4 sequencing reads.

**Table 1:** Summary of CSF examinations

Test items	Results	Normal range
Appearance	Clear	Clear
Color	Colorless	Colorless
Coagulum	Not present	Not present
Leukocyte count	1/µL	–
Red blood cell count	3/µL	–
Total protein	0.5	0.15–0.45
Albumin (mg/L)	349	< 350
Immunoglobulin G (mg/L)	49.90	< 34.0
Immunoglobulin A (mg/L)	9.99	< 5.0
Immunoglobulin M (mg/L)	0.67	< 1.3
Glucose (mmol/L)	7.5	2.5–4.5
Chlorine (mmol/L)	131	120–132
Lactate dehydrogenase (U/L)	19	–
Adenosine deaminase (U/L)	0	≤45
Microbial culture	Negative	Negative

Therefore, a diagnosis of probable COVID-19 associated encephalitis was considered instead of autoimmune encephalitis or epileptic seizures. We got a consultation with physicians from the department of infectious diseases, and adjusted anti-infection therapies to nirmatrelvir/ritonavir and cefotaxime. Meanwhile, considering the potential neuroinflammatory responses associated with COVID-19 encephalitis, the patient received a 1 g pulse dose of methylprednisolone followed by a 5-day course of immunoglobulin therapy. Following the administration of immunomodulatory therapy, the patient’s psychiatric symptoms improved significantly, and body temperature fluctuated around 37 °C. He was maintained on a low-dose oral hormone regimen, which was gradually tapered following discharge. Follow-up MRI performed one month after discharge revealed that the left subdural hematoma had been largely resolved. By the 3rd month, MRI from other hospital suggested resolution of the lesion with associated ventricular reduction. Unfortunately, the patient did not return for subsequent follow-up with our institution, and no repeat CSF testing or formal cognitive re-assessment was performed.

## Discussion and conclusion

In most reported cases, patients could present both respiratory symptoms and manifestations of COVID-19-associated encephalitis simultaneously. However, in our case, the patient initially exhibited only a mild fever without definitive respiratory symptoms. Although subsequent laboratory tests showed elevated IgG antibodies to SARS-CoV-2, both the naso-oropharyngeal swab and blood culture for SARS-CoV-2 detection were negative on admission. Typically, SARS-CoV-2 ribonucleic acid (RNA) tended to be either undetectable or very low levels in CSF samples from COVID-19 patients [3]. The limited detection possibilities of CSF SARS-CoV-2 might be attributed to the restricted sensitivity of conventional methods such as polymerase chain reaction (PCR) and pathogen culture. In our case, we applied sensitive mNGS method, which could identify a broader range of pathogens with a lower detection threshold. Specifically, a total of approximately 92.7 million raw reads were obtained, with a very low percent of low-quality reads (0.1%). After removing host sequences and simple repetitive sequences, a total of 4,515,181 pieces of data were acquired. For the detected SARS-CoV-2 sequences, which exhibited 100% relative abundance at the species level in the microbial composition analysis, the breadth of genome coverage reached 56.18%, supported by 4 high-quality reads uniquely mapped to the SARS-CoV-2 genome. Although the limited sequencing depth precluded definitive assessment of coverage across highly variable genomic regions, the substantial breadth of coverage (56.18%) provided strong genomic evidence supporting both specificity of detection and the validity of the SARS-CoV-2 positive finding in CSF samples. Confirmation tests such as reverse transcription-polymerase chain reaction (RT-PCR) tests, could help reduce the potential interference, for example, background pollution and misjudgment of cross reaction in mNGS measurement. In addition, the quantitative data from RT-PCR test could, to a certain extent, help assess the severity of the disease and evaluate the treatment effect. However, in our case, confirmatory testing for SARS-CoV-2 in the cerebrospinal fluid was not performed, as such follow-up confirmation was not routinely conducted in the department of infectious diseases unless additional clinical indications were present.

Some studies have analyzed the clinical characteristics of RNA-CSF-positive versus RNA-CSF-negative patients, finding that the presence of SARS-CoV-2 in CSF was more likely to induce encephalitis-associated neurological syndromes and result in typical psychiatric manifestations [4]. The exact pathogenesis of COVID-19-associated encephalitis remains under investigation, and several potential mechanisms have been proposed. First, SARS-CoV-2 has been shown to directly invade the central nervous system (CNS), cross the blood–brain barrier (BBB), and disrupt neuronal function. In addition, secondary neuronal injury might result from cytokine storm, hypoxia, molecular mimicry, or micro-thrombosis [5–7]. The hypothesis of autoimmune associated mechanism was supported by evidences of anti-neuronal and anti-glial antibodies in plasma or CSF samples [8]. In our case, the test results of the CSF showed mildly elevated total protein level, IgG and IgA, along with normal counts of white blood cells and red blood cells, which ruled out the possibility of sample contamination and was consistent with many reported cases of COVID-19-associated encephalitis. The CSF mNGS results indicated the exclusive positive infectious agent of SARS-CoV-2, suggesting viral involvement of the central nervous system. The other features supporting the diagnosis of probable COVID-19-associated encephalitis were summarized as follows: (1) the clinical presentation with psychiatric symptoms, cognitive dysfunction, and EEG abnormalities (diffuse slow waves) was consistent with encephalitis criteria; (2) negative autoimmune encephalitis antibody panels; (3) the favorable response to combined antiviral (nirmatrelvir/ritonavir) and immunomodulatory therapy (methylprednisolone and IVIG), suggesting the simultaneous presence of infectious and immunological factors. It could be quite difficult to distinguish direct viral invasion and para-infectious immune-mediated mechanisms in this patient. The term “COVID-19-associated encephalitis” was considered as a broader diagnostic category that could encompass both pathogenic mechanisms.

It was estimated that intracranial hemorrhage occurred in approximately less than 2% of all COVID-19 cases [9]. Under conditions of SARS-CoV-2 infection, endothelial cells could be targeted and induced to release pro-inflammatory cytokines [10]. Coagulopathy conditions were also affected in COVID-19 patients. In addition, elevated intra-abdominal and intrathoracic pressure, generally caused by common coughing symptoms, could synergistically contribute to cell dysfunction and hemorrhagic tendency. In our case, the etiology of subdural hematoma might be attributed to the following plausible mechanisms: (1) trauma-related: the patient exhibited violent psychiatric behaviors including “striking the head against the ground,” which could have directly caused subdural hemorrhage; (2) coagulopathy: although the patient’s coagulation parameters (INR, PT, and APTT) were within normal range, the elevated D-dimer level suggested an underlying prothrombotic/coagulopathic state, which was commonly seen in COVID-19 patients and might predispose to hemorrhagic complications; (3) endothelial dysfunction: SARS-CoV-2-induced endothelial injury and pro-inflammatory cytokine release (as evidenced by elevated IL-6 and TNF) could have contributed to vascular fragility. Based on the medical history, clinical presentation, and related examinations, the subdural hematoma was reasonably considered a concomitant finding with plausible etiologies, rather than a hemorrhagic complication directly related to COVID-19. Furthermore, for patients with altered mental status and subdural hematoma, non-convulsive status epilepticus (NCSE) is an important differential diagnosis. In our case, EEG monitoring showed diffused 3–6 Hz slow wave discharges with low amplitudes symmetrically in both hemispheres, without epileptiform discharges (such as sharp waves, spike-wave complexes, or periodic patterns) that would suggest NCSE. In addition, the patient’s psychiatric symptoms did not respond to the initial empirical anti-infection treatment but improved significantly after immunomodulatory therapy, further supporting an inflammatory/immune-mediated etiology over seizure-related pathology.

Given the potential neuroinflammatory responses associated with COVID-19 encephalitis, nirmatrelvir/ritonavir and immunomodulatory therapies, including corticosteroids and intravenous immunoglobulins, were administered in this case. Corticosteroids are recommended as first-line treatment [11]. Plasmapheresis, on the other hand, is considered more suitable for COVID-19-associated autoimmune encephalitis [12]. The combinations of immunoregulation therapies were also conducted in several case reports [13, 14]. Rituximab administration was suggested to relief severe neurological complications for COVID-19 patients [15]. For our patient, we ultimately implemented a multifaceted therapeutic strategy involving nirmatrelvir/ritonavir for SARS‑CoV‑2 infection, along with high‑dose methylprednisolone pulse therapy followed by intravenous immunoglobulin to address potential COVID‑19‑related neuroinflammatory responses.

In this report, we introduced a case of COVID-19-associated encephalitis and concomitant subdural hematoma. We recommend that the possibility of COVID-19-associated encephalitis should be considered in patients presenting with fever and psychiatric symptoms, even when initial investigations provide insufficient evidence of SARS-CoV-2 infection. Sensitive pathogen detection methods, such as metagenomic next‑generation sequencing and subsequent confirmatory testing, are essential for identifying potential infections. In addition, for COVID-19 patients presenting with neuropsychiatric symptoms and potential head trauma, prompt cranial imaging is suggested to identify possible hematoma. In summary, given the complexity and severity of COVID-19-associated encephalitis, clinicians should strive to achieve timely diagnosis, administer appropriate treatment, provide early prognostic assessment, and conduct dynamic follow-up for affected patients.

## Data Availability

All data and materials are available from the corresponding author upon request.

## Abbreviations

APTT: Activated partial thromboplastin time
BBB: Blood–brain barrier
CNS: Central nervous system
COVID-19: Coronavirus disease 2019
CRP: C-reactive protein
CSF: Cerebrospinal fluid
CT: Computerized tomography
EEG: Electroencephalogram
ESR: Erythrocyte sedimentation rate
Ig: Immunoglobulin
IL: Interleukin
INR: International normalized ratio
MRI: Magnetic resonance imaging
mNGS: Metagenomic next-generation sequencing
NCSE: Non-convulsive status epilepticus
PCR: Polymerase chain reaction
PT: Prothrombin time
RNA: Ribonucleic acid
RT-PCR: Reverse transcription-polymerase chain reaction
SARS-CoV-2: Severe acute respiratory syndrome-coronavirus-2
TNF: Tumor necrosis factor
